# 

**Published:** 1915-09

**Authors:** 


ATLANTA, GA.

PUBLISHED MONTHLY. ATLANTA SUBSCRIPTION $1.00

### JOURNAL-RECORD OF MEDICINE

Successor to { Atlanta Medical and Surgical Journal, Established 1855. and Southern Medical Record, Established 1870. } 62nd Year

Vol. LXII ATLANTA, GA., SEPTEMBER, 1915 No. 6

